# The Hydroxyurea Absorption Phenotype: A Key PK/PD Determinant in Sickle Cell Disease Treatment

**DOI:** 10.3390/pharmaceutics18060654

**Published:** 2026-05-27

**Authors:** Amelia-Naomi Sabo, Charlotte Nazon, Catherine Paillard, Véronique Kemmel

**Affiliations:** 1 Laboratoire de Biochimie et Biologie Moléculaire, Pôle de Biologie-Génétique-Pathologie, Hôpitaux Universitaires de Strasbourg, 67098 Strasbourg, France; veronique.kemmel@chru-strasbourg.fr; 2 Laboratoire de Pharmacologie et Toxicologie Neurocardiovasculaire, Unité de Recherche 7296, Faculté de Médecine de Maïeutique et des Sciences de la Santé, Centre de Recherche en Biomédecine de Strasbourg (CRBS), Université de Strasbourg, 67085 Strasbourg, France; 3 Centre de Compétence Pour les Maladies Constitutionnelles du Globule Rouge et de L’érythropoïèse, Service D’hématologie Oncologie Pédiatrique, Pôle de Pédiatrie, Hôpitaux Universitaires de Strasbourg, 67200 Strasbourg, France; charlotte.nazon@chru-strasbourg.fr (C.N.); catherine.paillard@chru-strasbourg.fr (C.P.); 4 Laboratoire D’immunoRhumatologie Moléculaire, INSERM UMR_S 1109, LabEx Transplantex, Fédération de Médecine Translationnelle de Strasbourg, Université de Strasbourg, 67085 Strasbourg, France

**Keywords:** hydroxyurea pharmacokinetics, sickle cell disease, hydroxyurea pharmacodynamics, absorption phenotype, therapeutic drug monitoring

## Abstract

**Background/Objectives**: Hydroxyurea (HU), a cornerstone treatment for sickle cell disease (SCD), exhibits marked interindividual pharmacokinetic/pharmacodynamic (PK/PD) variability that remains poorly understood. This study aimed to establish a population PK model using OPTIMDREP randomized trial data (NCT06464458), quantify parameter variability, and identify covariates influencing HU PK and hematological response. **Methods**: Plasma sampling data from 22 SCD patients (20 pediatric and 2 adult patients; median age: 11.2 years [range: 2.5–35.7]) on once-daily oral HU underwent a non-compartmental analysis (NCA) followed by nonlinear mixed-effects modeling (MonolixSuite^®^). PK variability covariates and PK/PD correlations with the mean corpuscular volume (MCV), reticulocytes, fetal hemoglobin percentage (HbF%) and neutrophils were investigated. **Results**: NCA identified two absorption phenotypes (rapid/slow), with higher maximum concentration values observed for rapid (36.5 ± 18.8 mg/L) compared to slow (22.3 ± 8.4 mg/L) (*p* = 0.0013) profiles but not statistically different total exposures, apparent clearances (Cl/F) or volumes of distribution (V_d_/F). The population approach identified the one-compartment model (first-order absorption and linear elimination) and confirmed the absorption phenotype as the key absorption rate (k_a_) covariate (9.93 vs. 1.36 h^−1^), explaining half of the k_a_ interindividual variability (IIV). The median-normalized body weight was retained for both the Cl/F and V_d_/F, as it significantly reduced the objective function value. No hematological parameter was correlated to PK parameters. However, rapid absorbers showed a superior response on the MCV (Δ = 9.1 fL, *p* < 0.0001), reticulocytes (Δ = −42.2 G/L, *p* < 0.01), and HbF% trend (Δ = 2.8%, *p* = 0.0835) but not on neutrophil counts (*p* = 0.8757). **Conclusions**: The absorption phenotype, a novel covariate explaining half of the k_a_ IIV, predicts a superior erythropoietic response without toxicity in SCD patients. These findings support absorption phenotype integration into PK-guided dosing algorithms to optimize early-response biomarkers and personalize HU therapy.

## 1. Introduction

Hydroxyurea (HU), also known as hydroxycarbamide, has been used for around 30 years in sickle cell disease (SCD), with proven long-term efficacy and a verified safety record [1]. In SCD, it prevents recurrent, painful vaso-occlusive crises and has been indicated in Europe since 2007 for patients aged ≥2 years old [2]. HU primarily induces fetal hemoglobin (HbF) production, which is normally prominent in fetal life but declines postnatally, with HbF (two α/two γ chains) improving oxygen affinity and red blood cell plasticity, thereby reducing vaso-occlusive crises, transfusion/hospitalization needs, and morbidity–mortality in SCD [3,4,5].

The initial dose recommended by the Food and Drug Administration (FDA) and the European Medicines Agency (EMA) is 15 mg/kg/day orally once daily, administered in the morning under fasting conditions, with regular hematological monitoring for toxicity. Dose escalation by 2.5–5 mg/kg/day every 3 months targets the maximum tolerated dose (MTD), which is between 14.2 and 35.5 mg/kg/day across patients, reflecting marked interindividual variability (IIV) driven by pharmacokinetic (PK) differences [6,7,8]. HU shows approximately 100% oral bioavailability, with a maximum plasma concentration (C_max_) of approximately 25 mg/L and a time to reach the C_max_ (T_max_) of approximately 1 h in adults. The drug displays wide tissue distribution, including blood–brain barrier penetration, and two well-described pediatric absorption phenotypes (rapid absorbers: T_max_ 15–30 min, C_max_ ~40 mg/L; slow absorbers: T_max_ 60–120 min, C_max_ ~20 mg/L) [9,10,11,12]. Despite these characteristics, interindividual exposure varies 5-fold in adults and 2–3-fold in children, prolonging dose escalation periods of 6–12 months, delaying benefits, and hindering adherence [11,13].

The pharmacodynamic (PD) response to HU is routinely monitored through a panel of hematological biomarkers. The HbF percentage reflects the degree of HbF induction and is the primary efficacy endpoint in most clinical trials [3,7]. The mean corpuscular volume (MCV) is a sensitive and early marker of HU activity, as erythroid progenitors exposed to HU produce larger red blood cells; it is therefore commonly used as a surrogate of HU-mediated erythropoiesis [14]. The absolute reticulocyte count decreases under HU treatment as a result of myelosuppression and reduced ineffective erythropoiesis, reflecting both drug activity and tolerance [3,7]. The absolute neutrophil count is the primary safety marker used to define the MTD, as excessive neutropenia is the main dose-limiting toxicity of HU [7,14,15]. Together, these biomarkers provide a comprehensive picture of HU efficacy and tolerability, yet their relationship with individual PK parameters, particularly the absorption phenotype, remains incompletely characterized in pediatric populations. Addressing this knowledge gap is critical to optimizing HU dosing strategies and reducing the time to MTD attainment in this vulnerable population.

This study aimed to establish a population PK model in SCD patients on HU, quantifying parameter variability and identifying key covariates. The kinetic profile emerged as a major covariate explaining both PK parameter variability and hematological PD endpoints related to HU compliance and MTD attainment.

## 2. Materials and Methods

### 2.1. OPTIMDREP Clinical Trial

Data were obtained from the prospective OPTIMDREP clinical trial (NCT06464458). Study information and a flow chart are available in the Supplementary Materials [16]. All patients or parents/guardians provided written informed consent before enrolment in this study. The study OPTIMDREP was designed to identify the most effective approach to reduce the time to reach the MTD of HU (Nazon et al., submitted) (Supplementary Table S1). Briefly, patients were randomized into two arms: arm A (control), in which dose adjustment was based on standard hematological monitoring, and arm B (experimental), in which dosing was guided by first-dose AUC measurement alongside hematological tolerance assessment, with the primary objective of reducing the time to reach the therapeutic MTD. In this study, the MTD was defined as neutrophil counts between 1.5 and 3.0 G/L and reticulocyte counts between 100 and 200 G/L at two consecutive visits under steady-state HU dosing [8,17]. Eligible patients were 2 to 35 years of age requiring therapeutic intensification for SCD or patients with poorly controlled HU treatment, defined as failure to reach the MTD. Arm A (control) consisted of a dose increase by 5 mg/kg/day every 3 months based on standard hematological monitoring, up to a maximum of 35 mg/kg/day. In arm B (experimental), an individualized PK-guided approach was applied: the individual area under the curve (AUC) measured at the first visit (V0) was used to estimate the dose expected to achieve the MTD based on the target AUC of 115 h.mg/L. The AUC target used for PK-guided dosing was derived from Dong et al., who established this threshold in a pediatric SCD population (median age: 8.8 years, range: 1.2–16.6 years) [6]. Dose adjustment was performed at visit 1 (V1, month 3). From V1 to visit 4 (V4), the two-hour post-dose concentration (C_2H_) was monitored but only hematological parameters were used to guide ongoing dose adjustments and assess tolerance. A second full AUC measurement was performed at visit 5 (V5).

Demographic information and standard laboratory parameters were collected at each visit from V0 to V5.

### 2.2. Blood Sampling

To obtain informative data, seven sampling times were prescribed at V0 and V5: before taking HU, and then 10 and 20 min and 1, 2, 4, and 6 h after taking the medication. From V1 to V4, samples were collected only at 2 h after HU intake. Blood samples were collected in 5 mL EDTA tubes. Whole blood was transported and/or stored at 2–8 °C for a maximum of 4 h before centrifugation at 1000 rpm for 10 min at 4 °C. Aliquoted plasma was rapidly frozen at −20 °C, and samples were analyzed once a week.

### 2.3. GC-MS Method

A volume of 50 µL of internal standard (tropic acid, 1 g/L in 0.9% NaCl) was added to 50 µL of patient plasma, quality control, calibrators and blanks (Etablissement Français du Sang, Strasbourg, France), followed by the addition of 1000 µL of a hexane/absolute ethanol mixture (50:50, *v*/*v*) to precipitate plasma proteins. The tubes were vortexed for 3 min and then centrifuged for 12 min at 2800× *g* and 4 °C. The supernatant was collected in new tubes and evaporated at 45 °C under a nitrogen flow. For the derivatization reaction, a BSTFA/heptane mixture (80:20, *v*/*v*) was prepared, and 100 µL was added under a fume hood to all tubes containing the dry extract. The tubes were incubated in an oven heated to 60 °C for 30 min. Once the tubes had cooled, they were centrifuged for 7 min at 4700 g and 4 °C. After transferring the mixture to vials suitable for GC-MS, 1 µL was injected into the analytical system (gas chromatography–mass spectrometry (GC-MS), ISQ LT, Thermo Fisher Scientific Inc., Waltham, MA, USA). The HU dosage method used a low-polarity RTX-5-MS capillary column (5% diphenyl/95% dimethyl polysiloxane, 30 m × 0.25 mm × 0.25 µm). Helium was the carrier gas. The initial injection temperature was 180 °C and the split mode was selected. The initial oven temperature was 80 °C (2 min), and then it increased by 12 °C/min to reach 170 °C and finally increased by 30 °C/min to reach 280 °C (2 min). The run lasted 15 min. The quantification and confirmation ions for HU were 277 and 292 m/z, respectively. The specific ion for tropic acid was 118 m/z. To calculate the HU concentration, the ratio of the HU peak area to that of the internal standard was used. The lower and upper limits of quantification were 0.79 mg/L and 100 mg/L, respectively. Method validation was performed in accordance with standard bioanalytical guidelines. The calibration curve was established in plasma from seven concentration points (2.5, 7.5, 10.0, 15.0, 20.0, 40.0, and 50.0 mg/L), with a coefficient of variation below 10% for all calibrators. Recovery, assessed as the ratio of the measured to added concentration, ranged from 99.6% to 105.6%, within the laboratory acceptance range of 90–110%. Intra- and inter-assay precisions were evaluated from 30 replicates at three concentration levels (7.5, 15.0, and 40.0 mg/L) and were below 15% at all levels, confirming satisfactory repeatability and reproducibility. Data processing was performed using Thermo Xcalibur^©^ software (version 2.2, Thermo Fisher Scientific Inc.).

### 2.4. PK Analysis

#### 2.4.1. Non-Compartmental Method

Plasma concentrations versus time were first assessed using a non-compartmental approach. Two PK profiles were identified based on the T_max_ at the first visit, consistent with those previously described [9,11]: rapid absorbers exhibited T_max_ values of 10 or 20 min (i.e., strictly below 1 h), and slow absorbers were defined by T_max_ values of 60 min or greater. A threshold of 1 h was therefore used to operationalize this distinction, whereby T_max_ < 1 h defined the rapid profile and T_max_ ≥ 1 h defined the slow profile. The AUC_0–6h_ was calculated using the trapezoidal rule with GraphPad Prism^®^ software (version 10.6.1). The AUC extrapolated to infinity (AUC_inf_) was computed as AUC_0–6h_ + C_6h_/λ_z_, where C_6h_ represents the last quantifiable plasma concentration post-HU administration and λ_z_ is the terminal elimination rate constant. This latter was estimated by the log-linear regression of the last two quantifiable concentration–time points of each individual profile. The apparent plasma clearance (Cl/F) and volume of distribution (V_d_/F) were derived from the AUC_inf_ and were also normalized to patient body weight (Cl/F/weight and V_d_/F/weight, respectively).

#### 2.4.2. Population Pharmacokinetics

Population pharmacokinetic modeling was performed to estimate typical population parameters (fixed effects), interindividual variability (IIV), and intraindividual variability (residual error). Concentrations $Y_{ij}$ for subject i at time j were modeled as:

(1) Y_ij_ = *f*(X_ij_, θ + η_i_) + ε_ij_

where *f* is the nonlinear structural model depending on covariates X_ij_; θ is the vector of the fixed-effect parameters; η_i_∼*N*(0,Ω) quantifies the IIV; and ε_ij_∼*N*(0,σ^2^) represents the residual error. Analysis used Monolix 2024R1, Simulations Plus with the Stochastic Approximation of the Expectation–Maximization (SAEM) algorithm for the maximum likelihood estimation of the θ, Ω and σ^2^. Empirical Bayes estimates of individual parameters employed the Hastings–Metropolis algorithm.

- Structural and Residual Error Model Selection

Base structural models were evaluated based on the parameter plausibility, precision, objective function value (OFV) (−2 log-likelihood), Likelihood Ratio Test (LRT), and residual error magnitude. Residual error models (additive, proportional, combined, exponential) were tested similarly. Parameter distributions (log-normal, probit-normal, power-normal) were selected if they significantly reduced the OFV.

- Covariate Selection

Covariates reducing IIV and improving goodness of fit (GOF) were identified via forward inclusion (ΔOFV > 3.84, *p* < 0.05, χ^2^, 1 degree of freedom) and backward deletion (ΔOFV > 7.88, *p* < 0.005, χ^2^, 1 degree of freedom), conservative thresholds for this small pediatric cohort to limit errors [18]. Selections were guided by pathophysiological/pharmacological rationale. Continuous covariates tested were age, body weight (BW), height, body mass index (BMI), serum creatinine, creatinine clearance [19], cystatin C, the cystatin C-based estimated glomerular filtration rate (eGFR) [20] and the treatment duration. Categorical covariates were sex, age (pediatric: 0 vs. adult: 1), randomization arm and kinetic profile (rapid or slow). Graphical exploratory analyses preceded testing. Missing data were handled as follows: categorical variables were coded as missing (.) in the dataset; continuous variables were imputed using population medians.

- Model Evaluation

Internal validation used LRT for nested models (ΔOFV vs. χ^2^ with degrees of freedom difference). Graphical diagnostics included: observed vs. population/individual predicted concentrations; normalized prediction distribution errors (NPDEs) vs. predictions/time; individual weighted residuals (IWRESs). Prediction-corrected visual predictive checks (pcVPCs) compared observed data distributions (median, 5th/95th percentiles) to 10,000 Monte Carlo simulations.

Model stability was assessed using a non-parametric bootstrap with 200 resamples generated by resampling individuals with replacement from the original dataset. Parameter estimates from each bootstrap replicate were compared to the original estimates to calculate bias (100 × [bootstrap median—original estimate]/original estimate) and 95% coverage probability (% of bootstrap 95% confidence intervals containing the original estimate). Acceptable model stability was defined as bias < ±15% for fixed effects and interindividual variability parameters.

### 2.5. Pharmacodynamic Analysis

Correlations between the population PK parameters and hematological criteria of MTD attainment (neutrophil and reticulocyte counts), efficacy (HbF percentage), and HU adherence (mean corpuscular volume (MCV)), were assessed using Pearson correlation tests. Associations between the kinetic profile and the HU efficacy, MTD attainment, and MCV were also evaluated.

### 2.6. Statistical Analysis

Data analysis was performed using MonolixSuite^®^, GraphPad Prism^®^, and Excel^®^ software. Statistical analyses and graphical representations were performed using GraphPad Prism^®^ (version 10.6.1). Non-compartmental analysis (NCA) of individual concentration–time profiles was performed using GraphPad Prism^®^. Population pharmacokinetic modeling, including covariate screening and model evaluation, was conducted using MonolixSuite^®^ (version 2024R1). Data management and calculation of the derived PK parameters (e.g., AUC_inf_, λ_z_) were performed using Excel^®^ (version Microsoft 365).

## 3. Results

### 3.1. Population Demographics

The study population consisted of 22 patients (14 males, 8 females), of whom 20 were under 18 years old and 2 were adult females (one aged 35.7 years and one aged 29.1 years), with a mean age of 12.2 ± 7.7 years, mean body weight of 35.8 ± 16.7 kg, and mean height of 141.5 ± 24.4 cm, reflecting a young and heterogeneous cohort in terms of growth. Among the 20 pediatric patients, the median age was 9.7 years (range: 2.5–16.4 years) and the median body weight was 31.0 kg (range: 11.4–60.0 kg). The HU daily dose was 20.3 ± 2.4 mg/kg, predominantly at steady-state (17/22), over a mean treatment duration of 4.5 ± 4.9 years, with a perfect balance between randomization arms A and B (11/11). Biological parameters indicated normal renal function (serum creatinine: 34.2 ± 7.7 µmol/L; cystatin C-based eGFR: 126.7 ± 14.2 mL/min/1.73 m^2^), moderately low MCVs (84.3 ± 12.0 fL), and mean fetal hemoglobin of 11.1 ± 6.3%, typical of a partial treatment response, also reflected by neutrophil (4.9 ± 2.4 G/L) and reticulocyte (240.9 ± 104.4 G/L) counts (Table 1).

### 3.2. Pharmacokinetic Analysis

A total of 44 pharmacokinetics profiles (obtained at V0 and V5) and 88 C_2H_ concentrations (obtained from V1 to V4) were analyzed, accounting for 391 observations.

#### 3.2.1. Non-Compartmental Analysis

**Figure 1:**
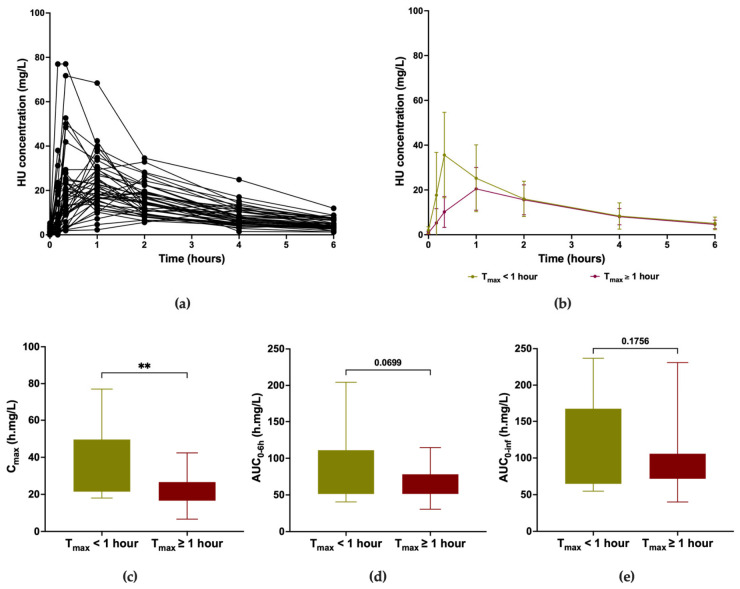
(**a**) Individual concentration–time hydroxyurea profiles (0–6 h post-dose) obtained at V0 and V5 showing marked interindividual variability. (**b**) Average concentration–time profiles showing rapid (T_max_ < 1 h, mustard-green line) vs. slow (T_max_ ≥ 1 h, purple line) kinetics. (**c**–**e**) Boxplots of key PK parameters according to kinetic profile: (**c**) C_max_, (**d**) AUC_0–6h_ and (**e**) AUC_inf_ showing significantly higher C_max_ values and a tendency to higher exposures for rapid profiles (mustard-green boxplots) compared to slow profiles (purple boxplots). Boxplots display median, interquartile range, and minimum–maximum values. Comparisons between two profile groups were performed using unpaired *t*-test. ** *p* < 0.01.

Non-compartmental analysis was performed for each individual. HU concentration–time profiles are presented in Figure 1a and show high IIV. Estimation of the C_max_ was 27.5 ± 14.7 mg/L (53.4%) (mean ± SD, coefficient of variation), with a T_max_ of 1.07 ± 0.89 h (81.9%). The AUC_0–6h_ and AUC_inf_ were estimated at 72.9 ± 32.5 mg.h/L (44.6%) and 101.2 ± 49.0 mg.h/L (48.5%), respectively. The estimated Cl/F, V_d_/F, Cl/F/weight and V_d_/F/weight were 9.1 ± 3.9 L/h (43.4%), 52.0 ± 45.5 L (87.5%), 0.26 ± 0.11 L/h/kg (43.0%) and 1.4 ± 1.1 L/kg (80.8%), respectively. Given the wide variability in the T_max_, the presence of two distinct pharmacokinetic profiles was investigated in the population, as previously described in the literature [11]. A total of 16 out of 44 profiles (36.4%) exhibited rapid kinetics, characterized by a T_max_ strictly <1 h, while the remaining profiles (63.6%) displayed T_max_ ≥ 1 h (Figure 1b). Five rapid absorbers were identified at V0 (one HU-naïve patient and four already on HU for a mean duration of 6.0 years) and 11 rapid absorbers at V5 (mean HU exposure: 7.3 years). Notably, two patients initially rapid at V0 developed slow profiles at V5. The kinetic phenotype (rapid or slow) statistically influenced the plasma HU concentrations, with higher C_max_ values observed for rapid profiles (36.5 ± 18.8 mg/L) compared to slow profiles (22.3 ± 8.4 mg/L) (*p* = 0.0013) (Figure 1c). Total exposure also showed a significant trend toward higher values in rapid profiles compared to slow profiles, although the difference was not statistically significant (*p* = 0.0699 and *p* = 0.1756 for the AUC_0–6h_ and AUC_inf_, respectively) (Figure 1d,e). No differences were observed in the Cl/F and V_d_/F, nor in their weight-normalized counterparts, between the two kinetic profiles.

#### 3.2.2. Population Pharmacokinetics

- Structural and Residual Error Model Selection

Nonlinear mixed-effects modeling was subsequently performed on the population data. A single-compartment model with first-order absorption, one compartment and linear elimination was initially tested to describe the data. However, the presence of delayed absorption in some patients orientated to a more flexible model. Thus, two different structural absorption models were tested and compared (Supplementary Table S2). Although the model with an absorption lag-time slightly improved the OFV (∆OFV = 22.42), it reduced the absorption rate constant precision (RSE = 39.9%) and increased its IIV. Choosing a transit compartment model yielded minimal gain (∆OFV = 5.38) with higher k_a_ imprecision. Adding a second compartment did not improve the predictive fit (Supplementary Table S2). Ultimately, the pharmacokinetic profiles were best described by a single-compartment model with first-order absorption and elimination. The residual error was best described by a combined additive (a = 1.60 mg/L) and proportional (b = 35%) model, and individual parameters followed a log-normal distribution, as selected based on their lowest OFV. IIV was moderate on the Cl/F (37.2%) and V_d_/F (49.3%) but high on the k_a_ (126.3%), reflecting variable absorption, likely due to different PK profiles in patients.

- Covariate Selection

**Figure 2:**
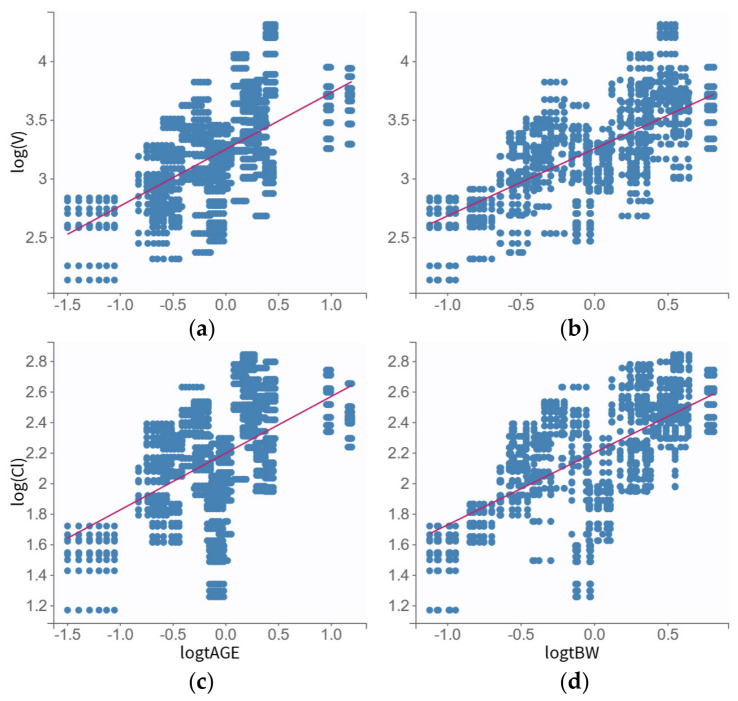
Correlation plots between individual empirical Bayes estimates (EBEs) of PK parameters and key covariates. Panels show log-transformed relationships: log(V_d_/F) (top row) vs. (**a**) log(median-normalized age) (r = 0.62) and (**b**) log(median-normalized BW) (r = 0.66); and log(Cl/F) (bottom row) vs. (**c**) log(median-normalized age) (r = 0.56) and (**d**) log(median-normalized BW) (r = 0.64). The red line represents the regression line.

The base population pharmacokinetic model without covariates estimated substantial IIV on all parameters. Following systematic covariate screening, the categorical covariate “rapid = 1” or “slow = 0” kinetic profile was identified as a significant predictor of the absorption rate constant (∆OFV = 44.61). Graphical relationships emerged between the Cl/F, V_d_/F and allometric covariates, such as age and BW, with strong positive correlations (r > 0.50) supporting covariate retention (Figure 2).

Once log-transformed median-normalized BW (NBW) was retained on the V_d_/F (∆OFV = 57.56) and Cl/F (∆OFV = 54.37), no other covariate statistically reduced the OFV. Creatinine covariate selection on the Cl/F did not sufficiently reduce the OFV and was not retained for the final model (∆OFV = 6.49). The incorporation of these covariates substantially reduced unexplained IIV, with final model estimates of 64.1% for the ka, 33.0% for the Cl/F and 34.7% for the Vd/F, compared to 126.3%, 37.2% and 49.3% in the base model, respectively. The allometric exponents for log-transformed NBW on the V_d_/F and Cl/F were estimated at 0.80 (RSE = 14.8%) and 0.64 (RSE = 13.9%), respectively, while the kinetic profile showed a strong categorical effect on the k_a_ with β_ka_KINETIC = 1.99 (RSE = 21.2%), corresponding to an approximately 7-fold increase in the absorption rate (ka_KINETIC = 1: 9.93 h^−1^ vs. ka_KINET = 0: 1.36 h^−1^). All covariate effects were precisely estimated with RSEs below 22%, confirming the robustness of the final covariate model (Table 2). A complete covariate screening table, including the ΔOFV and associated LRT *p*-values for all covariates tested on each parameter, is provided in Supplementary Table S3.

Thus, the formulas for estimating the k_ai_, Cl/F_i_ and V_d_/F_i_ of HU for a patient (i) with a body weight (BW_i_) according to this final model are:

(2) $${\mathrm{ka}}_{i}={ka}_{\mathrm{pop}}*{exp(\beta_{ka}}^{KINETIC=0\;or\;1})*\exp\left(\eta_{\mathrm{ka}}\right)$$

(3) $$V_{d}/F_{i}={Vd}_{\mathrm{pop}}*{\left(\frac{BWi}{34.9}\right)}^{\beta \_Vd\_NBW}*exp(\eta_{\mathrm{Vd}/F})$$

(4) $$\mathrm{Cl}/F_{i}={\mathrm{Cl}}_{\mathrm{pop}}*{\left(\frac{BWi}{34.9}\right)}^{\beta \_Cl\_NBW}*exp(\eta_{Cl/F})$$

The final model was composed of a one-compartment structural model with a combined error model. All parameters (k_a_, Cl/F, V_d_/F) followed a log-normal distribution. NBW was included as a covariate on the Cl/F and Vd/F and the kinetic profile on the k_a_.

- Model evaluation

**Figure 3:**
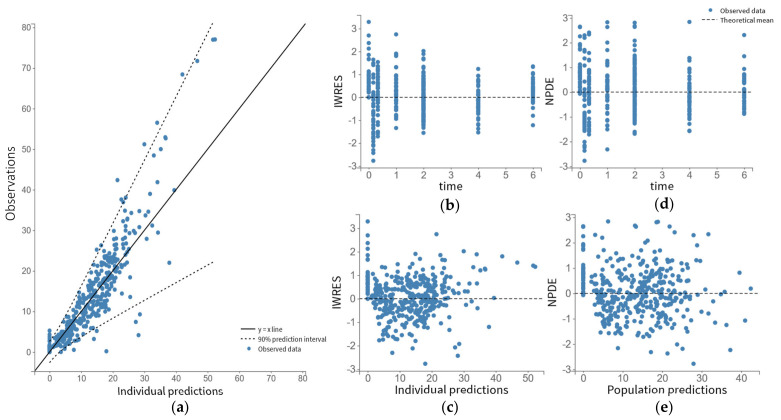
Plots illustrating model validation: (**a**) goodness-of-fit (GOF) plot showing adjustments between individually predicted values and observations; (**b**,**c**) individual weighted residual (IWRES) and (**d**,**e**) normalized prediction distribution error (NPDE) plots versus (**b**–**d**) time and (**c**–**e**) predicted concentrations.

**Figure 4:**
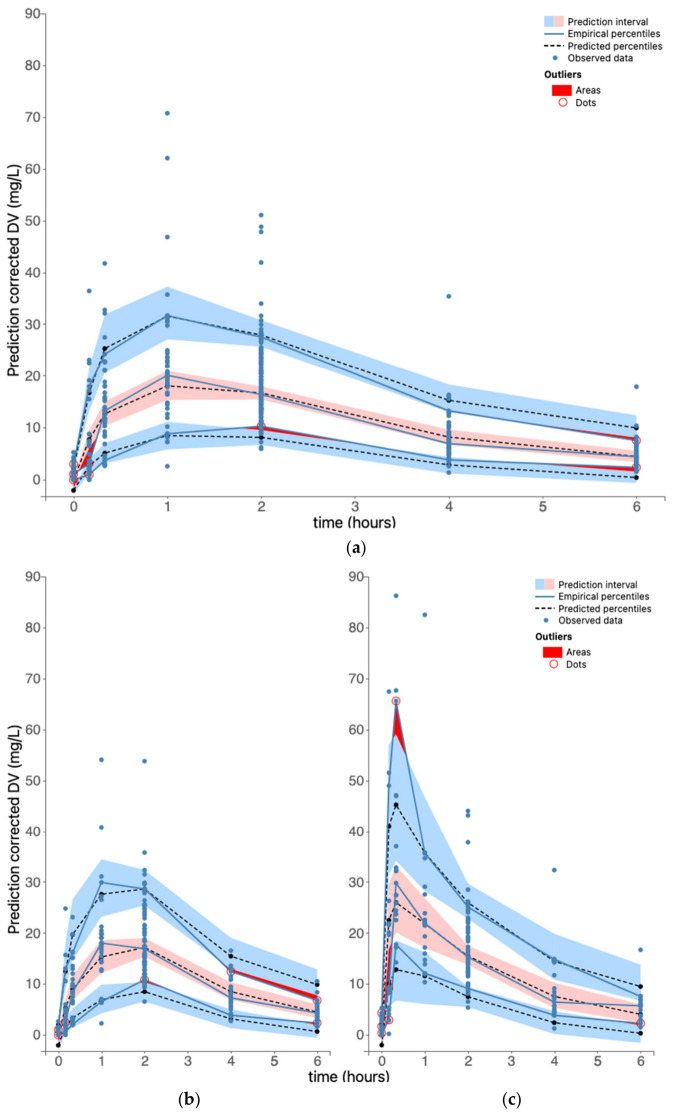
Prediction-corrected visual predictive checks (pcVPCs) for (**a**) all individuals; (**b**) slow kinetic profile (KINET = 0) and (**c**) rapid kinetic profile (KINET = 1), demonstrating model adequacy.

GOF plots show the homogeneous distribution of observed concentrations around the line of identity (y = x) for individual predictions (Figure 3a). IWRESs were primarily within the −2 to +2 range, evenly distributed around zero (Figure 3b,c). NPDEs followed a standard normal distribution without prediction- or time-dependent bias (Figure 3d,e), confirming model adequacy. Prediction-corrected VPCs (pcVPCs) demonstrated a very good model performance, with observed data distributions closely matching simulated percentiles across all timepoints, except minor deviations at 10 min and 2 h post-HU intake (Figure 4a). Split pcVPCs by categorical covariate KINETIC confirmed the adequate capture of interindividual variability, showing outliers after 5 h for slow profiles (KINET = 0) (Figure 4b) and the slight underestimation of absorption predictions for rapid profiles (KINET = 1) (Figure 4c).

Bootstrap (*n* = 200) confirmed parameter stability (bias < 15%), with the RSE of the bootstrap equivalent to the RSE of the initial model (Table 2).

### 3.3. Pharmacodynamic Analysis

No correlation was found between the population PK parameters (k_a_, Cl/F and V_d_/F) and hematological criteria for MTD attainment (neutrophil or reticulocyte counts), efficacy (HbF percentage), or HU adherence (MCV) (Table 3).

**Figure 5:**
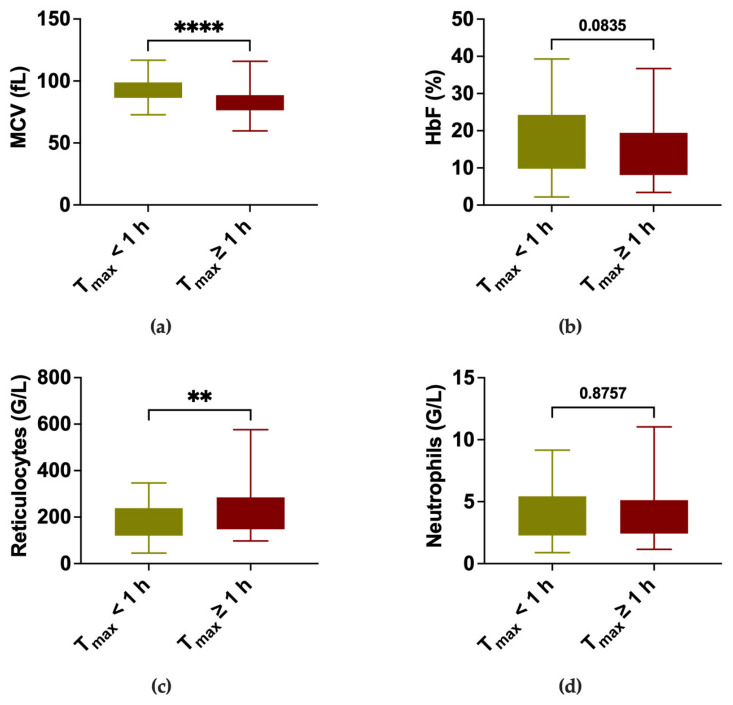
Boxplots of hematological parameters of patient compliance to HU treatment (mean corpuscular volume (MCV) (**a**), HU efficacy (HbF percentage) (**b**) and MTD attainment ((**c**) reticulocytes and (**d**) neutrophils) by kinetic profile (rapid (T_max_ < 1 h, mustard-green boxes) vs. slow (T_max_ ≥ 1 h, purple boxes). Boxplots display median, interquartile range, and minimum–maximum values. Comparisons between two profile groups were performed using unpaired *t*-test with Welch’s correction. ** *p* < 0.01; **** *p* < 0.0001.

Rapid kinetics (T_max_ < 1 h) was associated with significantly higher MCVs (93.4 ± 10.9 fL vs. 84.3 ± 11.731 fL, *p* < 0.0001, Figure 5a) and a strong trend towards higher HbF percentages (16.7 ± 9.89% vs. 13.9 ± 8.04%, *p* = 0.0835, Figure 5b) compared to slow absorption (T_max_ ≥ 1 h). Interestingly, reticulocytes were significantly lower in rapid kinetics patients (185.2 ± 75.5 G/L vs. 227.4 ± 105.2, *p* < 0.01, Figure 5c), while neutrophils showed no difference (*p* = 0.8757, Figure 5d). The MTD did not differ significantly between absorption phenotypes, although it showed a non-significant trend toward higher values in slow absorbers (*p* = 0.1662). These results support a PK-PD relationship where faster absorption enhances HU hematological efficacy and optimizes MTD hematological markers in this population.

## 4. Discussion

In this study, HU pharmacokinetics were first characterized using non-compartmental analysis, followed by population modeling. Multiple covariates explained PK parameter variability, among which the kinetic profile (slow vs. rapid absorption) emerged as a key determinant, significantly accounting for interindividual variability and correlating with PD parameters of HU compliance and tolerability.

HU is now recognized as a disease-modifying treatment for SCD. However, both in adults and children, this therapy exhibits substantial IIV in the required dose, efficacy, tolerability, and time to reach the MTD. Several studies suggest that therapeutic drug monitoring could reduce the time to achieve therapeutic targets or MTD by at least 3 months [6,7,11,21].

First, a non-compartmental analysis was performed for each individual. The mean Cl/F, V_d_/F, Cl/F/weight and V_d_/F/weight were 9.1 L/h, 52.0 L, 0.26 L/h/kg and 1.4 L/kg, respectively. The mean AUC_0–6h_ and AUC_inf_ were estimated at 72.9 mg.h/L and 101.2 mg.h/L, respectively. These values are consistent with those reported in non-compartmental studies. Specifically, mean AUC and clearance values of 102 mg.h/L and 7.98 L/h [9] and 107.3 mg.h/L and 9.32 L/h [22], respectively, have been previously described in the literature. Furthermore, Ware et al. [11] first identified two distinct absorption phenotypes, characterized by a T_max_ of 15–30 min in fast absorbers and of 60–120 min in slow absorbers; these findings were subsequently confirmed by Wiczling et al. [9]. The present study corroborates these observations, as two subpopulations were identified: one with a T_max_ strictly below 1 h, and a second with a T_max_ of 1 h or greater. The absorption phenotype (rapid vs. slow) significantly influenced the plasma HU concentrations, with rapid absorbers exhibiting higher C_max_ values (36.5 ± 18.8 mg/L) compared to slow absorbers (22.3 ± 8.4 mg/L). The impact of the absorption phenotype on the C_max_ observed in the present study is consistent with the seminal findings of Ware et al. [11], where rapid absorbers exhibited a significantly higher C_max_ (28.9 ± 6.9 mg/L) compared to slow absorbers (22.2 ± 4.3 mg/L; *p* < 0.001), along with a shorter mean residence time. Regarding total drug exposure, a trend toward higher AUC values was observed in rapid absorbers compared to slow absorbers, although this difference did not reach statistical significance, consistent with previously published data. Ware et al. reported that the absorption phenotype significantly influenced first-dose measures of systemic exposure; however, no detailed AUC values stratified by phenotype were provided in their publication [11]. This finding was further corroborated by Wiczling et al., who confirmed that the absorption profile does not significantly affect overall drug exposure [9].

This study subsequently characterized PK parameters and their variability using a population approach. The model best describing the observed data was a one-compartment model with first-order absorption and elimination. The presence of delayed absorption in some patients has been previously reported [10,11]; however, the inclusion of transit compartments or an absorption lag-time to describe the absorption phase did not meaningfully improve the parameter precision in the present analysis. Given the imprecision of parameter estimates associated with these alternative models in the present dataset, the one-compartment model with first-order absorption was retained as the most parsimonious and clinically interpretable structural model. A linear elimination model was retained, which is consistent with the PK behavior commonly described at the low doses of HU used in SCD. Indeed, several preclinical and clinical studies have demonstrated that at higher doses (20–80 mg/kg), such as those employed in hematological malignancies, HU elimination becomes saturable and requires Michaelis–Menten kinetics to be adequately characterized [10,22]. Beckloff et al. further proposed that an AUC/C_max_ ratio below 4 is indicative of linear elimination, whereas a ratio above 6 suggests saturable Michaelis–Menten kinetics [23]. In the present cohort, the calculated AUC/C_max_ ratio was approximately 2.8 and 4.5 for the rapid and slow PK profiles, respectively, thereby supporting and consolidating the choice of a linear elimination model.

The estimated V_d_/F, normalized to body weight, was 25.23 L for a typical patient weighing 34.9 kg, with an IIV of 34.7%. This value is consistent with those reported in the literature, ranging from 0.480 to 0.901 L/kg [10,13,22,23]. Apparent clearance was estimated at 9.06 L/h (IIV = 32.99%) for the same typical patient, in good agreement with the literature values in children, adolescents and adults with SCD [6,9,10]. A comparison of population PK parameter estimates from the present study with those reported in prior HU population PK studies in patients with SCD is summarized in Table 4. While the final model demonstrated satisfactory goodness-of-fit and internal validation metrics, external validation in larger, multicenter datasets encompassing broader demographic and geographic diversity will be required before these findings can be generalized to the wider SCD pediatric population. Following systematic covariate screening, the categorical covariate “rapid” (coded 1) vs. “slow” (coded 0) absorption phenotype was identified as a significant predictor of the absorption rate constant and was retained in the final model, as it accounted for approximately half of the interindividual variability in this parameter. The typical k_a_ value was 1.36 h^−1^ in slow absorbers, while it was 9.93 h^−1^ in rapid absorbers, reflecting a nearly 7-fold difference in the absorption rates between the two phenotypes. To the best of our knowledge, no published population pharmacokinetic model has previously incorporated the absorption phenotype as a significant covariate on the k_a_, making this a novel contribution of the present work.

Significant IIV was found for the Cl/F and V_d_/F. As this study enrolled predominantly pediatric patients, the selection of appropriate allometric scaling covariates was a critical step in model building. Log-transformed NBW and age yielded comparable results during covariate screening; however, BW is generally preferred in the literature [6,9,13]. Although age showed promising results, it fails to account for underweight or overweight status, as well as growth retardation, which are clinical conditions that are not uncommon in children with SCD. Allometric exponents of 0.64 and 0.80 on the weight/34.9 ratio were found to best predict individual Cl/F and V_d_/F values based on typical population values, a finding close to the exponents in the literature [6,13]. No other demographic, disease-related, or biological covariate tested reached the threshold for inclusion in the final model. A weak correlation was observed between serum creatinine and drug clearance (r = 0.50, *p* = 0.0299); however, the inclusion of this covariate did not meaningfully improve the model fit or reduce the IIV. Contrary to the findings first reported by Dong et al. [6], no significant relationship was identified between serum cystatin C and HU clearance in the present cohort. In their study (*n* = 96), the range of serum cystatin C concentrations was considerably wider (0.57–1.25 mg/L) and included children with impaired renal function. In contrast, all patients in the present study had normal renal function, which likely accounts for the absence of a detectable relationship between cystatin C and HU clearance.

Finally, the present study provides novel evidence of a PK-PD relationship between the absorption phenotype and hematological response to HU. Rapid absorbers demonstrated significantly higher MCV values and lower reticulocyte counts compared to slow absorbers, with a trend toward higher HbF percentages, collectively suggesting the enhanced erythropoietic efficacy of HU in this subgroup. These findings are in partial agreement with those reported by Ware et al. in the HUSTLE cohort [11], where the fast/slow absorption showed only a borderline association with the HbF percentage at the MTD (*p* = 0.0982, R^2^ = 0.05). Crucially, however, Ware et al. exclusively used the HbF percentage as their PD endpoint and did not investigate the relationship between the absorption phenotype and other key hematological parameters, namely, the MCV, reticulocyte count, or neutrophil count. The present study therefore extends these findings by demonstrating, for the first time, that rapid absorbers exhibit significantly higher MCV values and lower reticulocyte counts compared to slow absorbers, with a trend toward higher HbF percentages, suggesting a broader and more comprehensive hematological benefit associated with the rapid absorption phenotype. However, these results should be interpreted as exploratory, as individual PK parameters were not significantly correlated with hematological endpoints in our cohort. This absence of significant correlation, despite significant between-group differences according to absorption phenotype, may appear paradoxical but can be reconciled by several complementary considerations. First, the limited sample size (*n* = 22) substantially reduces statistical power for continuous PK-PD correlation analyses, while binary group comparisons retain greater sensitivity in small cohorts. Second, the PK-PD relationship for HU may follow a threshold rather than a linear model, whereby erythroid progenitor suppression reaches a plateau beyond a critical C_max_, attenuating continuous correlations while preserving group differences. Third, the absorption phenotype appears to reflect a predominantly stable individual characteristic, as supported by the absence of significant differences in the phenotype distributions between HU-naive and chronically treated patients. However, phenotype switching was observed in two patients who transitioned from a rapid to a slow profile between the first and fifth visit, indicating that the absorption phenotype is not entirely fixed and may be subject to intraindividual variability over time, possibly related to intercurrent clinical factors, adherence, or other patient-specific determinants. Despite this, rapid absorbers likely maintain higher daily C_max_ values throughout the entire course of treatment, generating a chronic cumulative pharmacological advantage that a single first-dose PK assessment cannot fully quantify, consistent with the S-phase-specific mechanism of HU cytotoxicity. Prospective studies incorporating repeated PK assessments will be required to characterize the long-term stability of the absorption phenotype and its implications for hematological response. Furthermore, the absence of a significant difference in the neutrophil counts between phenotypes is noteworthy. Two hypotheses may explain these findings. First, frequent infections in pediatric SCD patients induce reactive neutrophilia, masking HU’s stabilizing effect on the neutrophil count. Second, myelosuppressive toxicity is not phenotype-dependent, and both rapid and slow absorbers can safely reach the MTD. The MTD is conventionally defined as a stable and tolerated dose achieving a target range of mild marrow suppression, most commonly determined by the absolute neutrophil count associated with other hematological parameters (reticulocyte count, platelets) [4,11,12]. The MTD was equivalently attained across phenotypes, confirming that both rapid and slow absorbers safely reach the therapeutic MTD despite a non-significant trend toward higher doses in slow absorbers. Substantial interpatient variability exists both in the MTD itself and in the percentage of HbF achieved, suggesting that PK, PD, and genetic factors all contribute to the phenotypic variability in HU response [11,12]. The absorption phenotype may therefore represent one important but not exclusive determinant of this variability. Larger cohort studies are warranted to elucidate its relative contribution alongside other covariates.

This study has several limitations that should be acknowledged. First, the sample size was modest, which may have limited the statistical power to detect subtle covariate effects, particularly for renal biomarkers such as cystatin C and creatinine, as well as weaker PK-PD associations, including the non-significant trend observed for HbF. Second, the cohort mainly included pediatric patients with preserved renal function, with only two adult participants, limiting the generalizability of our findings to older patients, individuals with impaired renal function, or more ethnically diverse populations. Third, it should be acknowledged that the absorption phenotype was derived from the same concentration–time dataset used for population PK model building, which introduces a potential circularity. Prospective validation of this covariate in an independent, larger multicenter cohort, ideally using a standardized first-dose PK sampling protocol, will be required to confirm its role as a robust and generalizable predictor of the k_a_ in pediatric SCD patients treated with HU. The phenotype switching observed in two patients highlights a potential limitation of single-assessment classification strategies in clinical practice. While a first-dose PK assessment remains a pragmatic and accessible tool for phenotype characterization, repeated PK evaluations at key timepoints during dose escalation may be warranted to capture intraindividual variability in absorption behavior and refine the clinical utility of phenotype-guided dosing strategies. Finally, adherence was not assessed using direct measures, and pharmacogenetic data were not available, both of which may have contributed to the observed interindividual variability.

## 5. Conclusions

This study established a population PK model for HU in SCD patients, quantifying the parameter variability and identifying the absorption phenotype as a key covariate on the k_a_. Non-compartmental analysis confirmed two distinct absorption phenotypes (rapid vs. slow), validated by the population modeling approach. Rapid absorbers showed superior hematological response without evidence of increased myelosuppression at equivalent MTDs, suggesting the potential value of the absorption phenotype in future dose individualization strategies aimed at optimizing HbF induction while minimizing toxicity.

## Figures and Tables

**Table 1:** Demographic characteristics of and main information on HU treatment at inclusion (V0).

Characteristics	Variables	Values
Demographics	Male/female (N)	14/8
	Age (years) (mean ± SD)	12.2 ± 7.7
	Body weight (kg) (mean ± SD)	35.8 ± 16.7
	Height (cm) (mean ± SD)	141.5 ± 24.4
	Body mass index (kg/m^2^) (mean ± SD)	16.8 ± 3.7
HU information	Daily dose (mg/kg) (mean ± SD)	20.3 ± 2.4
	Initiation/steady-state (N)	5/17
	Duration of treatment (years) (mean ± SD)	4.5 ± 4.9
	Randomization arm A/arm B (N)	11/11
Biological parameters (mean ± SD)	Mean corpuscular volume (fL)	84.3 ± 12.0
	Reticulocytes (G/L)	240.9 ± 104.4
	White blood cells (G/L)	9.6 ± 3.2
	Neutrophils (G/L)	4.9 ± 2.4
	Platelets (G/L)	296.9 ± 82.9
	Serum creatinine (µmol/L)	34.2 ± 7.7
	Creatinine clearance (mL/min/1.73 m^2^)	176.5 ± 33.5
	Cystatin C (mg/L)	0.7 ± 0.1
	Cystatin C-based eGFR ^1^ (mL/min/1.73 m^2^)	126.7 ± 14.2
	Fetal hemoglobin (%)	11.1 ± 6.3

^1^ Cystatin C-based estimated glomerular filtration rate (eGFR) [20].

**Table 2:** Population pharmacokinetic parameter estimates of hydroxyurea from final Monolix model and bootstrap validation (*n* = 200).

	Parameter	Definition	Model Estimation (RSE)	Bootstrap Mean (RSE)|Bias (%)
	k_a_ (h^−1^)	Absorption rate constant	1.36 (14.80)	1.30 (18.2)|−4.52
	Cl/F (L/h)	Apparent HU clearance	9.06 (4.71)	9.17 (4.97)|+1.26
	V_d_/F (L)	Apparent volume of distribution	25.23 (7.69)	24.30 (5.71)|−3.68
Covariates	β_ka_KINETIC	Effect of kinetic profile on k_a_	1.99 (21.20)	1.86 (19.2)|−6.59
	β_Cl_NBW	Effect of body surface on Cl/F	0.64 (13.90)	0.65 (12.62)|1.26
	β_V_d__NBW	Effect of normalized BW on V_d_/F	0.80 (14.80)	0.80 (13.2)|0.44
	IIV-ka (%)	k_a_ interindividual variability	64.07 (22.20)	61.91 (19.69)|−2.73
	IIV-Cl/F (%)	Cl/F interindividual variability	32.99 (16.8)	32.00 (17.43)|−3.74
	IIV-V_d_/F (%)	V_d_/F interindividual variability	34.73 (18.10)	33.22 (18.12)|−6.85
Residual error	a	Additive residual error	1.60 (8.88)	1.61 (12.34)|0.51
	b	Proportional residual error	0.35 (6.33)	0.34 (6.88)|−0.78
OFV	−2 log-likelihood value	Objective function value	2408.21	

**Table 3:** Correlations between population pharmacokinetic (PK) parameters and hematological criteria of maximum tolerated dose attainment (neutrophil and reticulocyte counts), as well as between efficacy (fetal hemoglobin (HbF%)) and HU adherence (mean corpuscular volume (MCV)).

PK Parameter	Variable	r^2^
k_a_ (h^−1^)	Neutrophil count (G/L)	0.005924
	Reticulocyte count (G/L)	0.005095
	HbF (%)	0.003571
	MCV (fL)	0.1108
Cl/F (L/h)	Neutrophil count (G/L)	0.02062
	Reticulocyte count (G/L)	0.001857
	Fetal hemoglobin (HbF) (%)	0.003380
	MCV (fL)	0.1185
V_d_/F (L)	Neutrophil count (G/L)	0.05015
	Reticulocyte count (G/L)	0.008357
	HbF (%)	0.01870
	MCV (fL)	0.1603
AUC (h.mg/L)	Neutrophil count (G/L)	0.01040
	Reticulocyte count (G/L)	0.0005165
	HbF (%)	0.005311
	MCV (fL)	0.1250

**Table 4:** Comparison of population pharmacokinetic parameter estimates across published hydroxyurea population pharmacokinetic studies in patients with sickle cell disease. BW—body weight; cpt—compartment; MM—Michaelis–Menten.

Study	Population	N	Structural Model	k_a_ (h^−1^) (RSE, %)	Cl/F (L/h) (RSE, %)	V_d_/F (L) (RSE, %)	Key Covariates
Present model	20 children and 2 adults; 34.9 kg (median BW, used for normalization)	22	One-cpt, first-order absorption, linear elimination	1.36	9.06	25.2	Kinetic profile, normalized BW
Paule et al. (2011) [13]	Adults	81	Two-cpt, first-order absorption	3.02	11.6	45.3 for 70 kg (central cpt)	-
Wiczling et al. (2014) [9]	Children; 30.7 kg (mean)	21	One-cpt, transit absorption, dual elimination	Mean absorption time: 0.22 h	6.88	21.8	BW
Dong et al. (2016) [6]	Children; 26.6 kg (median)	96	One-cpt, MM elimination, transit absorption	8.19	19.56 for 70 kg	49.6 for 70 kg	BW, cystatin C

## Data Availability

The data presented in this study are available on request from the corresponding author. Data available on request as additional analyses from the same clinical trial are currently being prepared for separate publication.

## Supplementary Materials

The following supporting information can be downloaded at: https://www.mdpi.com/article/10.3390/pharmaceutics18060654/s1, Text S1: Sample Size Calculation; Text S2: Randomization; Figure S1: CONSORT 2025 Flow Diagram; Table S1: Description of the main and secondary objectives and their outcome measures. Table S2: Comparison of tested models during population PK model development; Figure S2: Eta-shrinkage of the individual parameters (conditional distribution) (a,c) and visual predictive checks (VPC) (b,d) plots of the 2 compartment-model tested (a,b) compared to the 1-compartment model (c,d); Table S3: Covariate screening results after graphical exploratory analyses and impact on interindividual variability. Ref. [16] cited in Supplementary Materials.

## Abbreviations

The following abbreviations are used in this manuscript:

AUC	Area under the curve
BMI	Body mass index
BW	Body weight
Cl/F	Apparent clearance
C_max_	Maximum plasma concentration
eGFR	Estimated glomerular filtration rate
GOF	Goodness of fit
HbF	Fetal hemoglobin
HU	Hydroxyurea
IIV	Interindividual variability
IWRES	Individual weighted residual
k_a_	Absorbance rate constant
LRT	Likelihood ratio test
MCV	Mean corpuscular volume
MTD	Maximum tolerated dose
NBW	Normalized body weight
NCA	Non-compartmental analysis
NPDE	Normalized prediction distribution error
OFV	Objective function value
pcVPC	Prediction-corrected visual predictive check
PD	Pharmacodynamics
PK	Pharmacokinetics
RSE	Relative standard error
SCD	Sickle cell disease
T_max_	Time to reach the maximum plasma concentration
V_d_/F	Apparent volume of distribution
