# Supplementary material for: The Hydroxyurea Absorption Phenotype: A Key PK/PD Determinant in Sickle Cell Disease Treatment
Source: Pharmaceutics. 2026 May 27;18(6):654. doi: 10.3390/pharmaceutics18060654 (PMC13305921; doi:10.3390/pharmaceutics18060654)
Supplement: Supplementary file 1 [file pharmaceutics-18-00654-s001.zip › pharmaceutics-4267695-supplementary.pdf]

Hydroxyurea Absorption Phenotype: A Key PK/PD Determinant in Sickle Cell Disease Treatment

Amelia-Naomi Sabo[1,2*], Charlotte Nazon[3], Catherine Paillard[3,4] and Véronique Kemmel[1,2]

[1] Laboratoire de Biochimie et Biologie Moléculaire, Pôle de Biologie-Génétique-Pathologie, Hôpitaux Universitaires de Strasbourg, 67098 Strasbourg, France; veronique.kemmel@chru-strasbourg.fr
[2] Laboratoire de Pharmacologie et Toxicologie Neurocardiovasculaire, Unité de Recherche 7296, Faculté de Médecine de Maïeutique et des Sciences de la Santé, Centre de Recherche en Biomédecine de Strasbourg (CRBS), Université de Strasbourg, 67085 Strasbourg, France; veronique.kemmel@chru-strasbourg.fr
[3] Centre de Compétence Pour les Maladies Constitutionnelles du Globule Rouge et de L'érythropoïèse, Service D'hématologie Oncologie Pédiatrique, Pôle de Pédiatrie, Hôpitaux Universitaires de Strasbourg, 67200 Strasbourg, France; charlotte.nazon@chru-strasbourg.fr (C.N.), catherine.paillard@chru-strasbourg.fr (C.P.)
[4] Laboratoire D'immunoRhumatologie Moléculaire, INSERM UMR_S 1109, LabEx Transplantex, Fédération de Médecine Translationnelle de Strasbourg, Université de Strasbourg, 67085 Strasbourg, France
* Correspondence: amelia-naomi.sabo@chru-strasbourg.fr; Tel.: +33-(0)-3-88-12-67-66

Supplementary Text S1: Sample Size Calculation

Sample size was determined using Bayesian simulations. In each group, the expected proportion of patients reaching the maximum tolerated dose (MTD) within 9 months ("success") was simulated using a design prior. Success rates were compared between groups using analysis priors, with power calculated as the proportion of simulations where the experimental arm (Arm B: AUC-guided dosing at Day 1 with hematological monitoring) showed superior success to the control arm (Arm A: hematological monitoring only).

Design priors:

- Arm A: Beta(90, 10)
- Arm B: Beta(10, 90)

Analysis priors:

- Arm A: Beta(1, 1)
- Arm B: Beta(1, 1)

With 10 patients per arm, power exceeds 95% for detecting a positive difference (lower bound of credible interval > 0). The probability that the posterior credible interval lower bound exceeds 20% is 86.5%. Empirical type I error rate (under equal success rates of 10-90%) is 3.65%. A 10-patient margin accounts for non-randomized inclusions and dropouts.

Supplementary Text S2: Randomization

Randomization was performed by the investigator or a delegated person 1:1 using permuted block randomization via the Cleanweb using personal access codes. Patients were allocated to one of the following treatment arms:

- Arm A (control): Dose adjustment based on standard hematological monitoring. In order to reproduce a usual care approach in the experimental arm A, the PK parameters (area under the curve-AUC and C2H) were not disclosed to the investigator in this arm.
- Arm B (experimental): Dose adjustment based on Day 1 AUC, followed by hematological tolerability assessment.

Supplementary Figure S1: CONSORT 2025 Flow Diagram

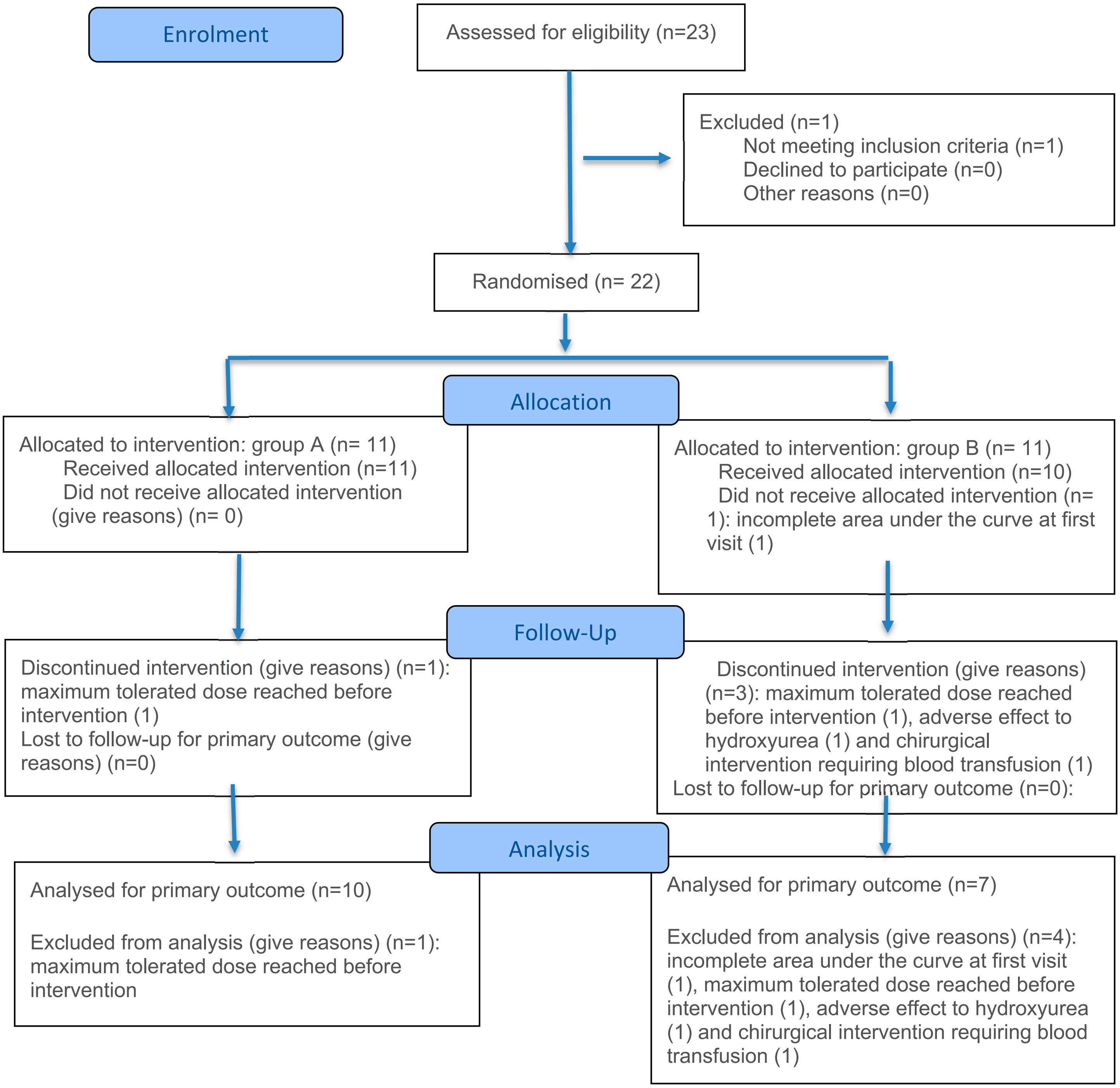

Supplementary Table S1: Description of the main and secondary objectives and their outcome measures

**Main objective**		
Comparison of the time to obtain the maximum tolerated dose (MTD) in 2 groups of sickle cell patients on hydroxyurea (HU) with different treatment follow-up methodology: MTD follow up or therapeutic pharmacological monitoring		- Arm A (Control): The dosage adjustment is quarterly from M3 onwards and will be made on hematological (WBC) tolerance. The increase of the dosage in HU will be 5 mg/kg every 3 months (or 2.5 mg/kg in case of renal insufficiency with ClCr ≤ 60 ml /min) until MTD is achieved and within a limit of 35 mg/kg/day (max 2500mg).<br><br>- Arm B (Experimental): Dose adjustment will be done at the M3 visit based on the results of the Pharmacokinetic analysis (area under the curve - AUC) performed on the day of the first HU intake after inclusion. At the quarterly visits V1 (3 months) to V4 (12 months), a HU assay at time T = 2H associated with monitoring of hematological monitoring of hematological tolerance will be performed to verify the pharmacokinetic stability in the child after the dosage adjustment.
**Secondary objectives**		
**1**	Assess the clinical efficacy of hydroxyurea treatment according to the two management strategies.	Clinical and Biological Efficacy Parameters (Both Arms): Number of vaso-occlusive crises; Number of sickle cell disease complications and/or hydroxyurea-related complications; Number of hospitalizations; Time to first blood transfusion; Fetal hemoglobin (HbF) percentage
**2**	Evaluate the tolerability of hydroxyurea treatment according to the two management strategies.	Biological Safety Parameters (Both Arms): Complete blood count, reticulocytes, ferritin; Renal function: Estimated glomerular filtration rate (eGFR) by cystatin C, plasma creatinine, urea; Liver function: AST, ALT, total and conjugated bilirubin
**3**	Conduct a pharmacokinetic/pharmacodynamic study of hydroxyurea in pediatric and adult populations.	PK Parameters (Both Arms): Area under the curve (AUC); Clearance (CL) and volume of distribution (Vd); Correlation between PK parameters and covariates
**4**	Build a population pharmacokinetic database and identify parameters contributing to hydroxyurea pharmacokinetic variability to better predict individualized dose adjustments from our study population.	Population pharmacokinetic modelling to identify covariates of inter- and intra-individual HU PK variability and improve individualized dose prediction
**5**	Confirm the rationale for reducing blood sampling in both children and adults by demonstrating that a single sample at 2 hours post-dose is sufficient to predict drug exposure and guide dose adjustment.	Pearson r^2 between HU concentrations at 2 hours post-dose and AUC

CONSORT 2025 checklist of information to include when reporting a randomised trial [1]

Section / Topic	No	CONSORT 2025 checklist item description	Reported on page no.
**Title and abstract**			
Title and structured abstract	1a	Identification as a randomised trial	1
	1b	Structured summary of the trial design, methods, results, and conclusions	1
**Open science**			
Trial registration	2	Name of trial registry, identifying number (with URL) and date of registration	2, 15
Protocol and statistical analysis plan	3	Where the trial protocol and statistical analysis plan can be accessed	2
Data sharing	4	Where and how the individual de-identified participant data (including data dictionary), statistical code and any other materials can be accessed	15
Funding and conflicts of interest	5a	Sources of funding and other support (e.g., supply of drugs), and role of funders in the design, conduct, analysis and reporting of the trial	15
	5b	Financial and other conflicts of interest of the manuscript authors	15
**Introduction**			
Background and rationale	6	Scientific background and rationale	2
Objectives	7	Specific objectives related to benefits and harms	2
**Methods**			
Patient and public involvement	8	Details of patient or public involvement in the design, conduct and reporting of the trial	2
Trial design	9	Description of trial design including type of trial (e.g., parallel group, crossover), allocation ratio, and framework (e.g., superiority, equivalence, non-inferiority, exploratory)	2
Changes to trial protocol	10	Important changes to the trial after it commenced including any outcomes or analyses that were not prespecified, with reason	NA
Trial setting	11	Settings (e.g., community, hospital) and locations (e.g., countries, sites) where the trial was conducted	15
Eligibility criteria	12a	Eligibility criteria for participants	2
	12b	If applicable, eligibility criteria for sites and for individuals delivering the interventions (e.g., surgeons, physiotherapists)	NA

Intervention and comparator	13	Intervention and comparator with sufficient details to allow replication. If relevant, where additional materials describing the intervention and comparator (e.g., intervention manual) can be accessed	2
Outcomes	14	Pre-specified primary and secondary outcomes, including the specific measurement variable (e.g., systolic blood pressure), analysis metric (e.g., change from baseline, final value, time to event), method of aggregation (e.g., median, proportion), and time point for each outcome	2 and supplementary material
Harms	15	How harms were defined and assessed (e.g., systematically, non-systematically)	2
Sample size	16a	How sample size was determined, including all assumptions supporting the sample size calculation	supplementary material
	16b	Explanation of any interim analyses and stopping guidelines	NA
Randomisation:			
Sequence generation	17a	Who generated the random allocation sequence and the method used	supplementary material
	17b	Type of randomisation and details of any restriction (e.g., stratification, blocking and block size)	supplementary material
Allocation concealment mechanism	18	Mechanism used to implement the random allocation sequence (e.g., central computer/telephone; sequentially numbered, opaque, sealed containers), describing any steps to conceal the sequence until interventions were assigned	supplementary material
Implementation	19	Whether the personnel who enrolled and those who assigned participants to the interventions had access to the random allocation sequence	supplementary material
Blinding	20a	Who was blinded after assignment to interventions (e.g., participants, care providers, outcome assessors, data analysts)	2, supplementary material
	20b	If blinded, how blinding was achieved and description of the similarity of interventions	supplementary material
Statistical methods	21a	Statistical methods used to compare groups for primary and secondary outcomes, including harms	3
	21b	Definition of who is included in each analysis (e.g., all randomised participants), and in which group	5
	21c	How missing data were handled in the analysis	4
	21d	Methods for any additional analyses (e.g., subgroup and sensitivity analyses), distinguishing prespecified from post-hoc	4
**Results**			
Participant flow, including flow diagram	22a	For each group, the numbers of participants who were randomly assigned, received intended intervention, and were analysed for the primary outcome	Supplementary material

	22b	For each group, losses and exclusions after randomisation, together with reasons	Supplementary material
Recruitment	23a	Dates defining the periods of recruitment and follow-up for outcomes of benefits and harms	2
	23b	If relevant, why the trial ended or was stopped	NA
Intervention and comparator delivery	24a	Intervention and comparator as they were actually administered (e.g., where appropriate, who delivered the intervention/comparator, how participants adhered, whether they were delivered as intended [fidelity])	2
	24b	Concomitant care received during the trial for each group	NA
Baseline data	25	A table showing baseline demographic and clinical characteristics for each group	5
Numbers analysed, outcomes and estimation	26	For each primary and secondary outcome, by group:<br>● the number of participants included in the analysis<br>● the number of participants with available data at the outcome time point<br>● result for each group, and the estimated effect size and its precision (such as 95% confidence interval)<br>● for binary outcomes, presentation of both absolute and relative effect size	5
Harms	27	All harms or unintended events in each group	NA
Ancillary analyses	28	Any other analyses performed, including subgroup and sensitivity analyses, distinguishing pre-specified from post-hoc	4
**Discussion**			
Interpretation	29	Interpretation consistent with results, balancing benefits and harms, and considering other relevant evidence	13-14
Limitations	30	Trial limitations, addressing sources of potential bias, imprecision, generalisability, and, if relevant, multiplicity of analyses	13-14

1. Hopewell, S.; Chan, A.-W.; Collins, G.S.; Hróbjartsson, A.; Moher, D.; Schulz, K.F.; Tunn, R.; Aggarwal, R.; Berkwits, M.; Berlin, J.A.; et al. CONSORT 2025 Statement: Updated Guideline for Reporting Randomised Trials. **2025**, doi:10.1136/bmj-2024-081123.

Supplementary Table S2: Comparison of tested models during population PK model development

Structural model	OFV -2 log-likelihood value	ΔOFV	k_a (h^{-1}) (RSE, %)	Cl/F (L/h) (RSE, %)	V_d/F (L) (RSE, %)	Additional parameters (value, RSE)
The administration is extravascular with a first order absorption (rate constant ka). The PK model has one compartment (volume V) and a linear elimination (clearance Cl).	2577.05	0	2.85 (26.4)	9.37 (8.95)	24.48 (12.7)	
The administration is extravascular with a first order absorption (rate constant ka) with a lag time (Tlag). The PK model has one compartment (volume V) and a linear elimination (clearance Cl).	2554.63	-22.42	6.87 (39.9)	9.01 (8.88)	27.07 (11.3)	Tlag = 0.11 (16.8)
The administration is extravascular with a first order absorption (rate constant ka) with transit compartments (mean transit time Mtt, transit rate Ktr). The PK model has one compartment (volume V) and a linear elimination (clearance Cl).	2571.67	-5.38	6.22 (556)	8.7 (15.6)	20.78 (31.7)	Ktr = 0.39 (5.24e+3)<br>Mtt = 0.46 (51.7)
The administration is extravascular with a first order absorption (rate constant ka). The PK model has a central compartment (volume V1), a peripheral compartment (volume V2, intercompartmental clearance Q), and a linear elimination (clearance Cl).	2571.63	-5.42	1.47 (135)	8.27 (13.9)	V1 = 14.31 (121)	Q = 4.67 (79.2)<br>V2 = 14.09 (71.2)
Final model	2408.21	-168.84	1.36 (14.80)	9.06 (4.71)	25.23 (7.69)	

Supplementary Figure S2: Eta-shrinkage of the individual parameters (conditional distribution) (a, c) and visual predictive checks (VPC) (b, d) plots of the 2 compartment-model tested (a, b) compared to the 1-compartment model (c, d)

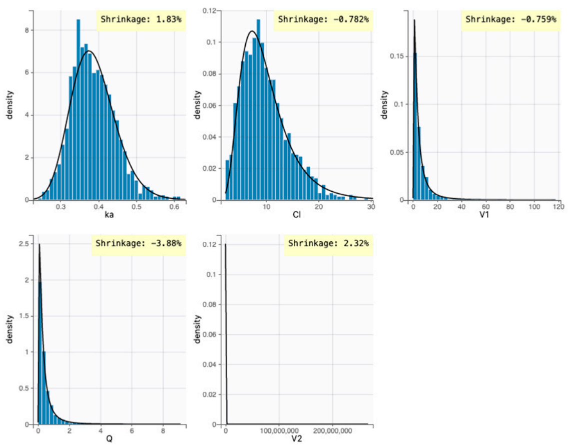

(a)

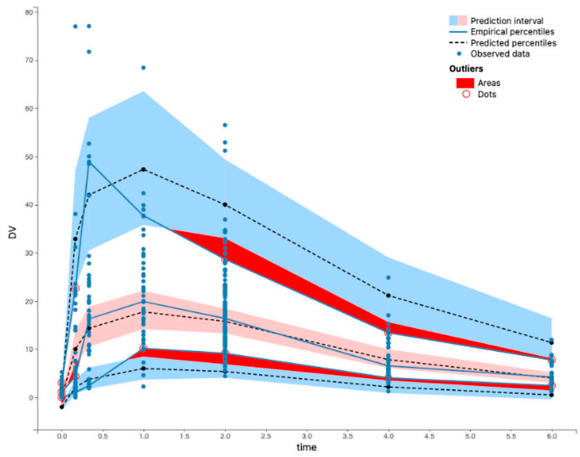

(b)

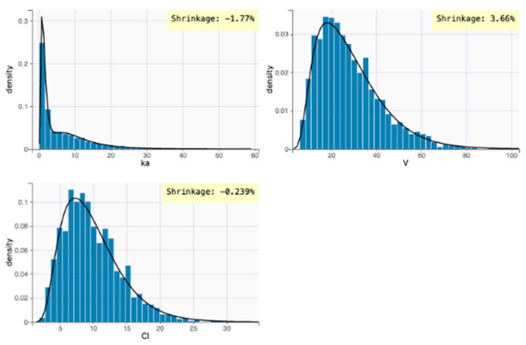

(c)

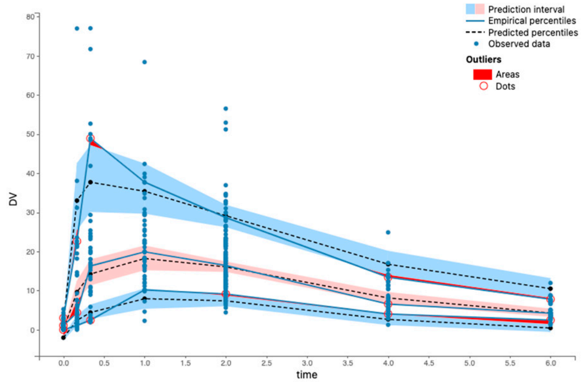

(d)

Supplementary Table S3: Covariate screening results after graphical exploratory analyses and impact on interindividual variability

**Covariate**	**Parameter**	**ΔOFV**	**LRT *p*-value**	**Decision**	IIV base model (CV%)	IIV final model (CV%)
Absorption phenotype	k_a	-44.61	*< 0.0001*	Retained	126.31	64.07
BW (log-normalized)	V_d/F	-57.56	*< 0.0001*	Retained	49.28	34.73
BW (log-normalized)	Cl/F	-54.37	*< 0.0001*	Retained	37.24	32.99
Creatinine	Cl/F	-6.49	*0.0299*	Not retained	-	-
Creatinine	V_d/F	-5.53	*0.0904*	Not retained	-	-
Sex	V_d/F	-1.34	*0.385*	Not retained	-	-
Sex	Cl/F	-1.18	*0.0138*	Not retained	-	-
Age (log-normalized)	V_d/F	0	*0.974*	Not retained	-	-
Age (categorial; 0 = pediatric; 1 = adult)	k_a	-0.23	*0.851*	Not retained	-	-
Age (categorial; 0 = pediatric; 1 = adult)	V_d/F	-0.67	*0.528*	Not retained	-	-
Age (categorial; 0 = pediatric; 1 = adult)	Cl/F	-0.37	*0.766*	Not retained	-	-
